# Panel-negative strategy for suspected fusion-driven papillary thyroid carcinoma

**DOI:** 10.1186/s13044-026-00301-x

**Published:** 2026-06-06

**Authors:** Yoichiro Okubo, Ikki Takada, Mei Kadoya, Soji Toda, Shuka Wada, Katsuhiko Masudo, Yohei Miyagi, Hiroyuki Hayashi

**Affiliations:** 1 Kanagawa Cancer Center, Department of Pathology, 2-3-2 Nakao, Asahi-ku, Yokohama, Kanagawa 241-8515 Japan; 2 Kanagawa Cancer Center, Department of Thoracic Surgery, Yokohama, Japan; 3 Kanagawa Cancer Center, Department of Endocrine Surgery, Yokohama, Japan; 4 Japanese Red Cross Medical Center, Department of Pathology, Tokyo, Japan; 5 Molecular Pathology and Genetics Division, Kanagawa Cancer Center Research Institute, Yokohama, Japan; 6 Department of Pathology, Yokohama Municipal Citizen’s Hospital, Yokohama, Japan

**Keywords:** Papillary thyroid carcinoma, Gene fusion, RNA-based fusion assay, Pan-TRK, Decision framework, Molecular diagnostics

## Abstract

Molecular testing in thyroid nodules and papillary thyroid carcinoma (PTC) is not required for every case, but it becomes clinically important when the result may influence diagnostic confidence, surgical planning, including the extent of surgery, or systemic therapy options. Representative scenarios include indeterminate thyroid cytology, clinically advanced or metastatic disease, and recurrent or progressive PTC. In these settings, the key question is not simply whether a common hotspot mutation is present, but whether clinically relevant drivers, including *RET* and *NTRK* fusions, have been adequately assessed. Fusion-inclusive molecular testing is preferable when molecular information is required for thyroid nodules or PTC, particularly when the clinical question includes the possibility of a fusion-driven tumor. However, in routine practice, limited or mutation-focused assays, such as *BRAF* V600E testing or hotspot DNA panels, may already have been performed because of limitations in access, reimbursement, specimen availability, or institutional workflow. Negative results from such assays can be misread as definitive exclusion of clinically relevant fusions, despite pre-analytic constraints and incomplete fusion coverage. This Correspondence does not advocate a hotspot-first workflow, nor does it propose serial add-on testing as a preferred strategy. Rather, we propose a pragmatic “panel-negative” communication and decision-making framework for situations in which limited testing has already been performed and morphologic or clinical suspicion for fusion-driven PTC persists. The approach emphasizes (i) selecting a thyroid-appropriate molecular assay from the outset whenever feasible, (ii) explicitly documenting residual suspicion and the scope of the performed assay in pathology reports, (iii) using pan-TRK immunohistochemistry only as an optional triage tool rather than a rule-out test, and (iv) considering RNA-based or other fusion-optimized assays only when the original clinical question remains unresolved and the result is clinically relevant. This framework is intended to reduce false reassurance from negative limited panels while reinforcing the need for appropriate molecular testing in the correct clinical scenario.

## Key points

Fusion-driven PTC may be missed if a negative panel is taken as definitive.

Morphology plus *BRAF* V600E negativity should trigger fusion gene workup.

Pan-TRK IHC is a screen, not a rule-out test; false negatives occur.

Specimen choice matters: avoid decalcified blocks; prioritize lymph node or separately fixed samples.

Reflex to RNA-based profiling when suspicion persists despite negative panels.

**Fig. 1:**
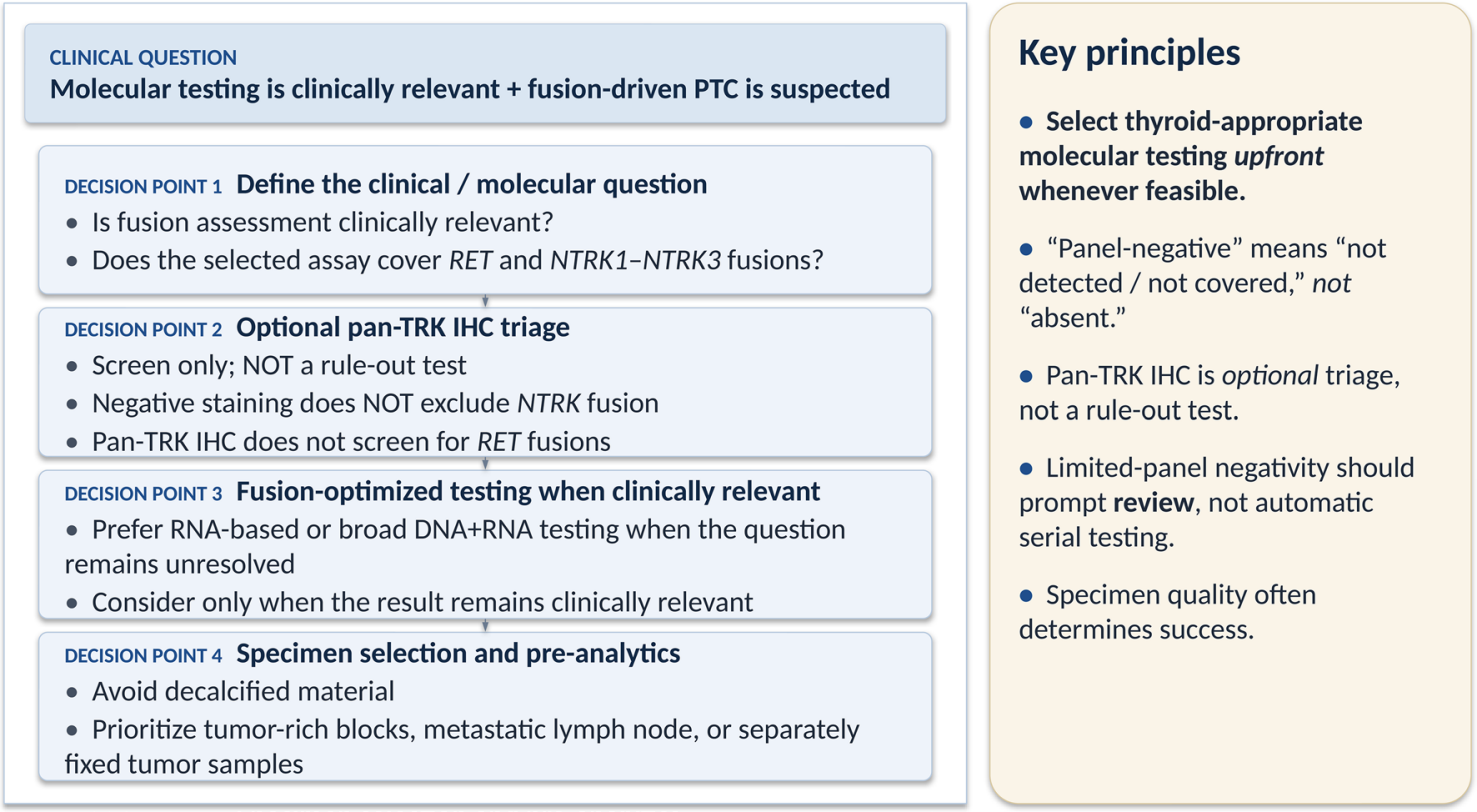
Decision framework for suspected fusion-driven papillary thyroid carcinoma after limited molecular testing. When molecular testing is clinically indicated for thyroid nodules or papillary thyroid carcinoma, the preferred approach is to select a thyroid-appropriate assay from the outset, including adequate assessment of clinically relevant fusions such as *RET* and *NTRK1–NTRK3*. This framework addresses the downstream situation in which limited or mutation-focused testing, such as *BRAF* V600E testing or a hotspot DNA panel, has already been performed. A negative result should prompt review of the assay scope, specimen quality, tumor content, and residual clinicopathologic suspicion rather than being interpreted as definitive exclusion of fusions. Pan-TRK immunohistochemistry may be used as an optional triage tool but should not be regarded as a rule-out test. When the clinical question remains unresolved and the result may influence surgical planning or systemic therapy, RNA-based or other fusion-optimized assays should be considered. PTC, papillary thyroid carcinoma; IHC, immunohistochemistry; TRK, tropomyosin receptor kinase

Papillary thyroid carcinoma (PTC) is a routine diagnosis in surgical pathology, but molecular testing is not required for every thyroid nodule or every PTC. Its clinical value depends on the scenario in which the result is expected to change diagnostic confidence, surgical planning, including the extent of surgery, or systemic therapy options. In indeterminate thyroid cytology, molecular testing may contribute to risk stratification and decisions regarding diagnostic lobectomy versus more extensive initial surgery. In clinically advanced, metastatic, recurrent, or progressive PTC, molecular testing may identify actionable alterations, including *RET* and *NTRK* fusions, that can inform systemic therapy selection [1]. When molecular testing is clinically indicated and fusion-driven PTC is within the differential diagnosis [1, 2], the preferred approach is to select a thyroid-appropriate assay from the outset, including adequate assessment of recurrent thyroid cancer drivers and clinically relevant fusions. Limited mutation-focused assays, such as *BRAF* V600E testing or hotspot DNA panels, may answer specific questions, but they should not be regarded as comprehensive tests for fusion-driven thyroid tumors. The present Correspondence is not intended to justify a hotspot-first workflow or an inefficient serial-testing strategy. Instead, it addresses a practical downstream problem that arises when limited or mutation-focused testing has already been performed because of local availability, reimbursement, specimen constraints [3], or institutional workflow. In such situations, a negative result may be incorrectly interpreted as excluding clinically relevant fusions. The goal of the “panel-negative” strategy is therefore to support accurate interpretation of assay scope, communication of residual suspicion, and appropriate escalation only when the original clinical question remains unresolved and the result is clinically relevant. A practical “panel-negative” framework can be organized around four decision points rather than mandatory sequential tests (Fig. 1):

## Decision point 1 — Define the clinical and molecular question before interpreting the test result

When molecular testing is requested or considered, the pathology report and clinical discussion should clarify the question being asked. For example, the purpose may be risk stratification in indeterminate cytology, support for surgical planning, or identification of actionable alterations in advanced, metastatic, recurrent, or progressive disease. If fusion-driven PTC is within the differential diagnosis, the selected assay should be evaluated for whether it adequately detects clinically relevant fusions, including *RET* and *NTRK1–NTRK3* fusions. If a limited assay has already been performed, explicit language such as “fusion not excluded” or “assay does not comprehensively evaluate fusions” can help prevent a negative hotspot panel from being interpreted as a definitive stop signal.

## Decision point 2 — Use pan-TRK immunohistochemistry only as an optional triage tool

Pan-TRK immunohistochemistry can be useful as a rapid adjunct when *NTRK* fusion is suspected and tissue or access to molecular testing is limited [2, 4–7]. However, it should not be positioned as a required intermediate step before molecular testing. Sensitivity and specificity vary by antibody clone, fixation, tumor type, and fusion partner. A positive result may support urgent confirmatory testing, but a negative result should not be used to rule out *NTRK* fusion when the pre-test probability remains high or when the clinical decision requires a definitive molecular answer [4–7].

## Decision point 3 — Use fusion-optimized assays when the clinical question requires fusion assessment

When the clinical question is whether an actionable fusion is present, the appropriate assay should be selected from the outset whenever feasible. RNA-based fusion assays or other fusion-optimized methods are generally better aligned with this question than mutation-focused hotspot panels [7, 8]. If a limited DNA panel has already been performed and is negative, escalation to a fusion-optimized assay should be considered only when the result remains clinically relevant, such as in advanced, metastatic, recurrent, or progressive disease, or when the morphology and clinical context strongly suggest fusion-driven PTC.

## Decision point 4 — Treat specimen selection and preservation as part of appropriate molecular testing.

Even when the correct assay is selected, false-negative or technically limited results may occur because of low tumor fraction, decalcification, prolonged fixation, or block selection that does not capture the relevant component [3]. When molecular testing is clinically anticipated, tumor-rich material should be prioritized, decalcified blocks should be avoided when possible, and macrodissection or alternative specimens such as metastatic lymph node deposits should be considered. These pre-analytic decisions are not secondary details; they are part of selecting an appropriate molecular testing strategy for thyroid tumors.

In summary, the central issue is not simply how to respond after a negative limited panel, but how to ensure that molecular testing is appropriate for the clinical question in thyroid tumors. When molecular information is expected to influence diagnosis, surgical planning, or systemic therapy, assays that adequately assess clinically relevant thyroid cancer drivers, including *RET* and *NTRK* fusions, should be selected from the outset whenever feasible. The “panel-negative” framework proposed here is intended as a practical safeguard for cases in which limited testing has already been performed, helping pathologists communicate assay scope, preserve appropriate tissue, and avoid false reassurance from negative results. It should not be interpreted as a recommendation for universal sequential testing in all *BRAF* V600E-negative PTCs.

## Data Availability

Data sharing is not applicable to this article as no datasets were generated or analyzed during the current study.

## Abbreviations

IHC: Immunohistochemistry
PTC: Papillary thyroid carcinoma
TRK: Tropomyosin receptor kinase
